# Visual Impairment and Ocular Findings in Children With Developmental Delay Attending a Child Development Unit Clinic at a Tertiary Hospital

**DOI:** 10.7759/cureus.95277

**Published:** 2025-10-23

**Authors:** Monika Singh, Miriam A Clement, Gowtham Kim, Gayathri J Panicker, Somreeta Bhattacharya, Radha Annamalai

**Affiliations:** 1 Department of Ophthalmology, Sri Ramachandra Institute of Higher Education and Research, Chennai, IND; 2 Department of Optometry, Faculty of Allied of Health Sciences, Sri Ramachandra Institute of Higher Education and Research, Chennai, IND

**Keywords:** developmental delay, exotropia, hypermetropia, ocular alignment, refractive error, strabismus, visual impairment

## Abstract

Introduction

Childhood blindness is largely attributable to preventable causes. In children with developmental delay, identifying ocular morbidity is particularly challenging due to limited cooperation and associated comorbidities. This study aimed to determine the prevalence and spectrum of refractive errors, strabismus, and other ocular findings among children with developmental delay attending a tertiary care center in South India, and to highlight the importance of early ophthalmic screening in this population.

Methods

This retrospective review included 100 preschool children with developmental delay (aged four months to five years) presenting to the ophthalmology outpatient department of a tertiary care center in South India over a period of two years. Visual impairment was defined as visual acuity below the age-appropriate expected level despite best correction, or, in uncooperative children, based on abnormal fixation behavior and inability to maintain central, steady fixation. Evaluation included visual acuity testing, refraction, ocular alignment assessment, and comprehensive ophthalmic examination.

Results

Visual impairment was observed in 66 (66%) children (95% CI: 56-75). Refractive errors were identified in 84% (95% CI: 75-90) of the cohort, with hypermetropia being the most frequent subtype (65, 78%), followed by myopia (15, 18%) and astigmatism (38, 45%). Strabismus was detected in 12% of children, most frequently exotropia. Causes of developmental delay included prematurity (69%), syndromic associations such as Down syndrome (10%), and miscellaneous factors (21%).

Conclusion

Nearly two-thirds of children with developmental delay had visual impairment, predominantly due to refractive errors and strabismus. Early ophthalmic screening and timely management are essential to reduce preventable visual disability and improve neurodevelopmental outcomes in this vulnerable group.

## Introduction

Childhood blindness and visual impairment constitute a major public health concern worldwide, with significant social, psychological, and economic consequences. In developing countries, it is estimated that 7% to 31% of childhood blindness and visual impairment is avoidable, 10% to 58% is treatable, and 3% to 28% is preventable [1]. Globally, approximately 1.4 million children are blind, and nearly 500,000 new cases are added each year [2]. In India, the prevalence of childhood blindness is reported to be 0.8 per 1,000 children in the age group of 0-15 years, amounting to nearly 280,000 blind children [3].

The control of blindness in children is of paramount importance because many causes of childhood blindness are also associated with high child mortality. Furthermore, children with vision loss face a lifetime of blindness, leading to a high cumulative burden of “blind years,” second only to cataract-related blindness in adults [3]. Beyond the medical impact, visual impairment profoundly affects a child’s psychological well-being, educational opportunities, and overall quality of life, often extending into adulthood [4].

Epidemiological data on childhood blindness and visual impairment are limited, particularly in children with developmental delays such as speech or language impairments. The rarity and heterogeneity of ocular disorders, combined with the difficulty of assessing vision in these children, pose significant methodological challenges [5]. Visual impairment in this population is especially concerning as it affects mobility, fine motor skills, communication, language development, self-care, and overall quality of life [6]. Importantly, such deficits often go unnoticed, either due to the child’s inability to recognize poor vision or because vision concerns are overshadowed by other health problems. Early screening is therefore essential to ensure timely detection and intervention [6].

Previous studies from South India have reported ocular findings in children with developmental delay, but the available literature remains scarce [6,7]. Variations in socioeconomic conditions and disparities in access to healthcare further complicate the analysis of this vulnerable group. Visual impairment in these children may result from a range of conditions, including cortical blindness secondary to perinatal hypoxia, birth asphyxia, periventricular hemorrhage, hydrocephalus, or meningitis. In addition, uncorrected refractive errors, cataracts, glaucoma, strabismus, amblyopia, ptosis, and congenital or acquired anomalies such as nasolacrimal duct obstruction, as well as systemic and syndromic associations, contribute to vision loss [6,7].

This study aimed to determine the prevalence and spectrum of visual impairment and ocular abnormalities, including refractive errors and strabismus, among children with developmental delay attending a tertiary care center in South India, and to highlight the importance of early ophthalmic screening in this population.

## Materials and methods

This retrospective study was conducted in the Ophthalmology Outpatient Department of a tertiary care hospital (Sri Ramachandra Institute of Higher Education and Research, Chennai) in South India from 2022 to 2024. The study population included preschool children aged 4 months to 5 years who presented with developmental delay in at least one domain (motor, social, or language development). A total of 100 consecutive children meeting the inclusion criteria were analyzed. Children with acute ocular trauma, prior ocular surgery, or incomplete records were excluded. The study protocol was approved by the Institutional Ethics Committee (CSP-MED/24/JAN/97/07). Written informed consent was obtained from parents or legal guardians of all participants, for open-access publication and identifiable clinical photographs.

Visual assessment

Visual Impairment was defined as visual acuity worse than age-appropriate expected levels: <20/60 for ages 4-12 months (Lea paddles), <20/40 for ages 1-3 years (Lea symbols or Landolt C), and <20/30 for ages 4-5 years [8]. For uncooperative children, fixation poorer than central/steady/maintained (CSM) was considered abnormal. Blindness was defined as visual acuity worse than 3/60 or equivalent fixation response.

Refraction and quality control

Refraction was performed using static retinoscopy, followed by cycloplegic retinoscopy using cyclopentolate or atropine as indicated. Post-cycloplegic findings were used for classification. All examinations were performed by a trained pediatric optometrist and verified by a consultant ophthalmologist to ensure inter-observer reliability. Refractive errors were defined as follows: hyperopia, ≥ +2.00 D spherical equivalent in either eye; myopia, ≤ −0.50 D spherical equivalent; astigmatism, ≥ 1.00 D cylinder; anisometropia, ≥ 1.00 D interocular difference; and amblyopia, ≥2-line interocular difference in best-corrected acuity or fixation preference with visual acuity below age norms [8].

Ocular alignment and orthoptic testing

Orthoptic assessment included motility testing (broad H test), cover test, near point of convergence, and fusional amplitude evaluation. Dynamic retinoscopy was used to assess accommodative response. Convergence insufficiency was defined as near point of convergence >10 cm and exophoria at near >6Δ greater than at distance, and accommodative insufficiency was defined as accommodative amplitude ≥2D below the age-expected norm (Hofstetter’s formula) [8].

Statistical analysis

Data were analyzed using IBM SPSS Statistics, version 30 (IBM Corp., Armonk, NY). Descriptive statistics were applied. Frequencies and percentages are reported with 95% confidence intervals (CIs). Confidence intervals were calculated using the Wilson method for binomial proportions.

## Results

Visual impairment, defined as acuity below the expected level for age, was observed in 66 (66%; 95% CI: 56-75) of children. Refractive errors were identified in 84% (95% CI: 75-90) of the screened cohort, with hypermetropia being the most frequent subtype, accounting for 65 (78%) cases. Myopia constituted 15 (18%) of the refractive error cases, and 38 (45%) of affected children had coexistent astigmatism (Table 1).

**Table 1:** Distribution of refractive errors among children with developmental delay (n = 84, with refractive error) Percentages do not total 100% as astigmatism may co-occur with hypermetropia or myopia. The range of spherical equivalents was +6.50 D to −11.00 D (median +1.75 D).

Refractive error (84 children)	Number of children (%)
		Hypermetropia	65 (78)
Myopia	15 (18)
Astigmatism	38 (45)

**Figure 1:**
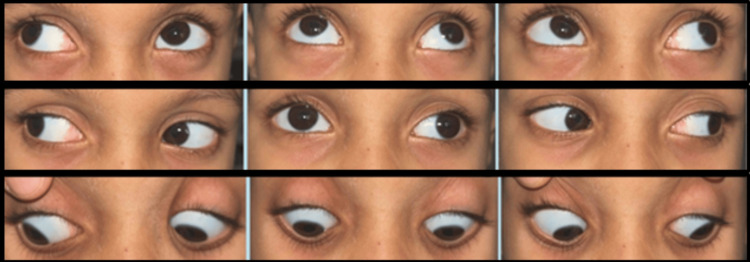
Nine gaze pictures showing a comitant left exotropia of 45 prism diopters (PD) and full extraocular movements in a five-year-old girl with global developmental delay

The maximum refractive errors recorded were +6.50 D, hypermetropia; −11.00 D, myopia; and 6.00 D, cylinder of astigmatism. Ocular misalignment was present in 12 (12%; 95% CI: 7-19) of children, with exotropia being the most common deviation (9, 9%), including two cases with associated vertical deviation (Figure 1).

**Figure 2:**
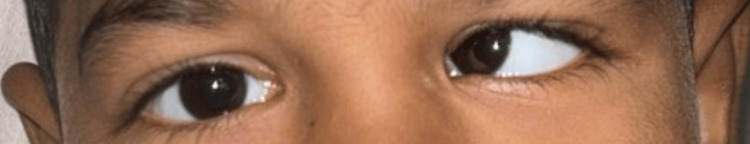
A 2.5-year-old boy with hypoxic ischemic encephalopathy sequelae with a large left comitant esotropia of 60 prism diopters (PD) with left dissociated vertical deviation and right-sided head tilt, and no significant refractive error

Esotropia was identified in three children, one of whom had dissociated vertical deviation (Figure 2).

Orthoptic evaluation revealed convergence insufficiency in 9 (9%) and accommodative insufficiency in 5 (5%). Amblyopia was detected in 15 (15%) of children, and nystagmus in 3 (3%). Additional ocular pathologies included bilateral congenital lamellar cataracts in one child and optic atrophy in five children (Table 2).

**Table 2:** Ocular findings in children with developmental delay (n = 100)

Ocular findings (100 children)	Number of children (%)
		Strabismus	12 (12)
Nystagmus	3 (3)
Amblyopia	15 (15)
Convergence insufficiency	9 (9)
Errors of accommodation	5 (5)
Congenital cataract	1 (1)
Optic atrophy	5 (5)

Incidental findings included Bitot’s spots, iris heterochromia, and allergic conjunctivitis. In children with Down syndrome, epicanthic folds and mongoloid slant were the most frequent ocular features, followed by blepharitis and nasolacrimal duct obstruction.

Ocular findings in 100 children were stratified by etiology of developmental delay: prematurity (69%), syndromic causes (10%), and miscellaneous (21%). Visual impairment was most frequent among syndromic children, while refractive error was predominant among those with prematurity.

Management strategies were tailored according to findings. Corrective spectacles were prescribed for children with significant refractive errors. Accommodative esotropia was managed with hyperopic correction, while children with large-angle deviations were referred for squint surgery following anti-suppression exercises and occlusion therapy for amblyopia.

## Discussion

Children with developmental disorders are at a higher risk of visual impairment than their neurotypical peers. Clinical examination in this group is often challenging due to poor cooperation, cognitive limitations, and associated systemic comorbidities. Early detection of ocular abnormalities is therefore essential, as unrecognized visual deficits may further hinder cognitive, motor, and social development [7,9].

In the present study, refractive error was the most common ocular morbidity among preschool children with developmental delay, with hypermetropia being the predominant subtype. This finding aligns with the results of Roch-Levecq et al. [4], who demonstrated that bilateral uncorrected hyperopia in preschool children was associated with reduced visuomotor integration. Similarly, Atkinson et al. [10] reported that hyperopia delayed visual-cognitive and visuomotor development. Astigmatism was the second most common refractive error observed, followed by myopia, consistent with earlier studies. Refractive errors are known to occur four times more frequently in preterm infants compared with those born at term [11], particularly among infants treated for retinopathy of prematurity [12].

The link between neurodevelopmental disorders and ocular morbidity is well established. Approximately 40% of children with autism spectrum disorder have ocular comorbidities, most commonly refractive error, strabismus, and amblyopia [13]. Children with Down syndrome are predisposed to multiple ocular pathologies, including anisometropia, reduced accommodation, strabismus, nasolacrimal duct obstruction, blepharitis, cataract, and keratoconus [14]. Cortical visual impairment, a common cause of vision loss in children with developmental delay, typically arises from hypoxic injury to the posterior visual pathways and leads to impaired visual processing [15].

The global burden of visual impairment due to uncorrected refractive error has risen steadily, with blindness attributable to this cause and the number of cases increasing from 6.3 million in 1990 to 6.8 million in 2010 [14]. By 2020, an estimated 160 million people had moderate to severe vision impairment due to uncorrected distance refractive error, and 510 million were affected by uncorrected near vision impairment [16]. Projections indicate that vision loss from uncorrected refractive error will increase by 55% by 2050, underscoring the need for timely screening and intervention [17].

Nearly three-quarters of children with neurodisabilities require ophthalmic intervention during their lifetime [18]. Strabismus and optic atrophy are particularly common in this group [19]. Among children with cerebral palsy (CP), ocular anomalies occur in 50%-90% of cases [20], with the highest prevalence of spastic CP [21]. Exotropia is especially frequent, with rates exceeding those in the general pediatric population [22]. If left untreated, ocular misalignment may lead to amblyopia, resulting in permanent visual disability and psychological distress [23].

The coexistence of epilepsy and genetic syndromes further increases the risk of ophthalmic disease in children with developmental delay [24]. Developmental delay in the visual domain, compounded by amblyopia, substantially contributes to overall disability [25]. In our study, in addition to refractive error, many children demonstrated ocular deviations, nystagmus, and optic atrophy, all of which were associated with significant visual morbidity. These findings emphasize the importance of routine and comprehensive ophthalmic screening in children with developmental disorders, as timely diagnosis and management can reduce the burden of visual disability and improve quality of life [26].

A limitation of this study is its retrospective design, which restricted the availability of detailed clinical and follow-up data. The relatively small sample size from a single tertiary care center may also limit the generalizability of the findings. Furthermore, variability in the level of cooperation among young children could have influenced the accuracy of visual acuity and orthoptic assessments. Inclusion of children from an ophthalmology OPD introduces a selection bias, likely overestimating ocular morbidity relative to community-based samples. Despite these limitations, the study provides important insights into the spectrum of ocular morbidity in children with developmental delay and highlights the need for systematic ophthalmic evaluation in this vulnerable population.

## Conclusions

This study highlights the considerable burden of visual impairment and ocular morbidity among children with developmental delay, with refractive errors being the most prevalent abnormality. Hypermetropia was the predominant subtype, followed by astigmatism and myopia, while a substantial proportion of children also presented with strabismus, amblyopia, and optic atrophy. The findings emphasize that uncorrected refractive errors and other treatable ocular conditions significantly contribute to visual disability in this vulnerable group. Incorporating early ophthalmic screening, prompt correction of refractive errors, and targeted management of strabismus and amblyopia into routine care is crucial to improve visual outcomes and promote optimal neurodevelopment in children with developmental disorders.
